# Exploring Immersion Coating as a Cost-Effective Method for Small-Scale Production of Enteric-Coated Gelatin Capsules

**DOI:** 10.3390/ph17040433

**Published:** 2024-03-28

**Authors:** Beatrice Sabbatini, Diego Romano Perinelli, Giovanni Filippo Palmieri, Marco Cespi, Giulia Bonacucina

**Affiliations:** School of Pharmacy, University of Camerino, 62032 Camerino, Italy; beatrice.sabbatini@unicam.it (B.S.); diego.perinelli@unicam.it (D.R.P.); gianfilippo.palmieri@unicam.it (G.F.P.); giulia.bonacucina@unicam.it (G.B.)

**Keywords:** Eudragit, hypromellose acetate succinate, paracetamol, tramadol HCl, enteric capsules, enteric coating, DRcaps^®^, Vcaps^®^ Enteric

## Abstract

The coating process for solid dosage forms is widely used in the pharmaceutical industry but presents challenges for small-scale production, needed in personalized medicine and clinical or galenic settings. This study aimed to evaluate immersion coating, a cost-effective small-scale method, for enteric-coated gelatin capsules using standard equipment. Two enteric coating polymers and different polymer concentrations were tested, along with API solubility. Results were compared with commercially available enteric capsule shells. Successful preparation of enteric coating capsules via immersion necessitates a comprehensive grasp of API and enteric polymer behavior. However, utilizing commercially available enteric capsule shells does not guarantee ease or robustness, as their efficacy hinges on the attributes of the active ingredient and excipients. Notably, coating with Eudragit S100 stands out for its superior process robustness, requiring minimal or no development time, thus representing the best option for small-scale enteric capsule production.

## 1. Introduction

The coating of a solid dosage form is a widespread process in the pharmaceutical industry because it offers a solution to many challenges encountered by scientists in the past. Indeed, there are various reasons for coating a dosage form [1,2]. Among these, coating can mask bad taste and odor [3,4], and it can protect the drug from oxygen, humidity, or other environmental factors [4,5]. The coating’s role can be aesthetic because it can improve the identification of the medicine (it has been proven that a colored film can aid in drug recognition, especially for elderly patients in polytherapy), and it can hide imperfections of the pharmaceutical form, particularly in the presence of APIs and excipients with different colors [6]. The coating can be also used to modify the drug release kinetics to obtain constant and prolonged release or ensure release in specific districts of the GI tract [7,8]. It is also possible, by combining coating with other modified release technologies, to obtain very complex release profiles, such as programmed ones [9]. One of the most common coatings used for modifying release applications is that applied to prevent the dissolution of the drug in the stomach and aim to release it in the intestine. This type of coating is usually called ‘gastro-resistant or enteric coating’, and it allows the administration of APIs targeted to the intestine or those that must avoid the stomach [10]. The enteric release of the drug is usually obtained by film coating using polymers with pH-dependent water solubility. Methacrylic acid–methyl methacrylate copolymers, cellulose derivatives with weak acid groups (such as cellulose acetate phthalate or hypromellose acetate succinate), or polyvinyl acetate phthalate are examples of polymers commonly used for enteric coating [1,2].

The industrial production of enteric film-coated dosage forms is a well-established process carried out using fluid bed or coating pan equipment. In almost all cases, the process involves the spray application of the coating solution and simultaneous drying, allowing to produce a large number of coated dosage forms in the same batch [1]. While industrial methods are highly effective for large-scale production, they are completely unsuitable for small batches, as required in personalized medicine and clinical production of coated dosage forms (e.g., hospital pharmacy or galenic preparation in a local pharmacy). The alternatives developed for these particular situations include two approaches: the use of capsule shells made of polymers having a pH-dependent dissolution or the design of inexpensive and easy-to-manage equipment that allows the coating of small batches. The first approach requires the use of the so-called Enteric Capsule Drug Delivery Technology (ECDDT). Despite these capsule shells having higher costs than traditional ones, they do not require additional equipment, and the preparations and formulations are exactly like the traditional ones. Several types of ECDDTs are now available on the market (such as Vcaps^®^ Enteric and DRcaps^®^) [11]. In addition, the development of new ECDDTs represents an interesting topic for researchers in the pharmaceutical area [12,13]. Despite them potentially being the ideal solutions, the literature results are controversial, and in many cases, failures in obtaining enteric release profiles were reported [14]. The second approach is represented by equipment based on the immersion coating procedure (such as the ProCoater^®^ by Torpac, Fairfield, NJ, USA), which allows the coating of small batches of oblong tablets or capsules by dipping these dosage forms into the coating solution [15]. These instruments enable the coating of a certain number of units simultaneously, and the coating solution can be used for several batches subsequently. However, dip coating presents several challenges for the formulator because the immersion of the dosage form in the coating solution is far more stressful than spraying or atomizing the solution on the pharmaceutical form. In addition, there are several variables to be optimized, such as the choice of the polymer, its percentage in the solution, the type and amount of plasticizer, the choice of solvents, and the formulation of the dosage form to be coated. Moreover, the success of the coating also depends on the process parameters, including the immersion time, the removal of excess liquid, and the type and duration of drying. Very few pieces of data are available for the process carried out through the immersion coating procedure, and most users rely on indications derived from studies performed with traditional spray-based methods. Among the published data, Moghimipour et al. [16] studied the effect of multiple coating layers of Eudragit FS 30D, an aqueous dispersion of methacrylic acid–methyl methacrylate copolymers, while Fülöpová et al. [17] evaluated the application of three types of enteric coating polymers on gelatin capsules or DRcaps^®^. Interestingly, the last study demonstrated that the coated gelatin capsules never achieved gastro-resistance, regardless of the type of polymer coatings and the concentration of the dispersions. In both cases, the coating process was not described.

The aim of this study was to assess the immersion coating of gelatin capsules using commercially available equipment to achieve standardized results. Two different grades of two enteric coating polymers were chosen, specifically Eudragit^®^ S100, Eudragit^®^ L100 (acrylic-based polymers), HPMC AS-MF, and HPMC AS-HF (cellulose-based polymers). Coating was carried out using organic polymer dispersions at two concentration levels. The capsules were filled with a formulation containing two model APIs with significantly different water solubility: paracetamol (AAP), defined as sparingly soluble by the European Pharmacopoeia (EP), and tramadol hydrochloride (Tram), reported by the EP as very soluble in water. The solubility of both drugs is pH-independent under the tested conditions (pKa of 9.38 and 9.41 for AAP and Tram, respectively [18,19]). The results were compared with those obtained through the ECDDT approach, using both single and cap-in-cap DRcaps^®^ or Vcaps^®^.

## 2. Results and Discussion

### 2.1. Coating Solution Rheology

The rheological analyses were performed to assess the rheological behavior of the prepared coating solutions and to explore variations among formulations with different polymers and varying percentages of the added polymer.

**Figure 1:**
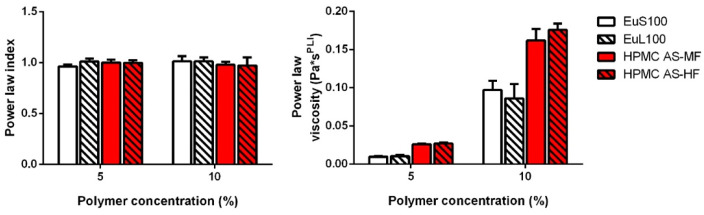
Effect of polymer type and concentration on the power law index (**left panel**) and the power law viscosity (**right panel**) as determined from a power law modeling of the viscometry data.

All the samples exhibited a power law index within the range of 0.96–1.01, indicating a linear increase in stress with the applied shear rate (Figure 1, left panel). Consequently, the samples displayed Newtonian behavior. The viscosity of the coating solution increased proportionally with the polymer content in the formulation. Specifically, formulations containing 10% polymer exhibited a viscosity approximately 8–10 times higher than those with a concentration of 5%. Samples prepared with cellulose derivatives demonstrated higher viscosity compared to Eudragit counterparts at all concentrations. However, no discernible differences were observed between samples containing Eudragit-type or cellulose-type polymers (Figure 1, right panel). In all cases, the viscosity of the samples consistently fell within the ideal range of 0.15–0.4 Pa*s for coating solutions applied with standard methods [20].

### 2.2. Dissolution Studies

**Figure 2:**
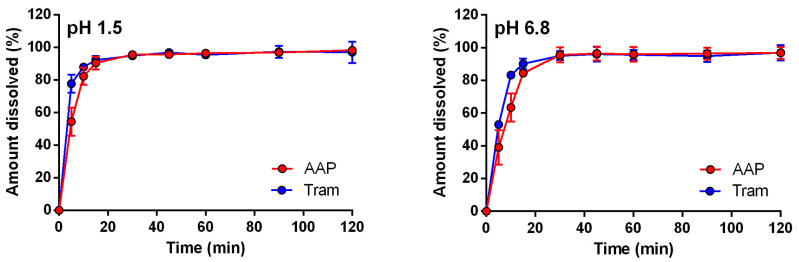
Release profiles of uncoated capsules containing AAP or Tram at pH 1.5 and 6.8.

The initial preliminary test conducted involved the dissolution of the uncoated capsules filled with the formulations of both drugs, AAP and Tram. The release rate of both APIs exhibited rapid release initially, reaching a semi-steady state around 20 min at pH 1.5 and 30 min at pH 6.8. This discrepancy at the two pH is consistent with the basic nature of the two drugs. Minimal differences were observed between the two APIs, particularly in the minutes before reaching the plateau, where Tram, owing to its higher water solubility, demonstrated a faster release (Figure 2).

#### 2.2.1. Capsules Coated with Eudragit Polymers

**Figure 3:**
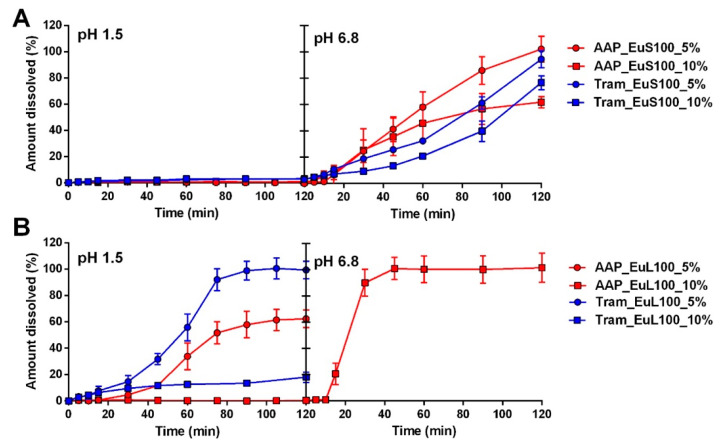
Release profiles of capsules containing AAP or Tram coated with EuS100 (**A**) and EuL100 (**B**).

The results of applying EuS100 at two concentrations for both AAP and Tram capsules are presented in Figure 3A. In all cases, the Eudragit S100 coating ensured an enteric release, with no significant drug release in the acidic medium, meeting the criteria for being considered gastro-resistant. However, differences were observed in the release kinetics in the intestinal-like fluid, dependent on the polymer concentration in the coating dispersion and the type of drug. Specifically, an increase in polymer concentration from 5% to 10% resulted in incomplete drug release after two hours (after two hours, the amount of drug release at pH 6.8 was around 60 and 70% for AAP and Tram, respectively). This aspect was more pronounced for AAP than for Tram, and it can likely be attributed to their different solubilities.

The results exhibited marked differences when EuS100 was replaced with EuL100 (Figure 3B). Specifically, at the lowest polymer concentration, the coating failed to prevent the dissolution of AAP or Tram in the acidic medium. Similarly, tramadol capsules coated with 10% EuL100 dispersion yielded the same outcome. In all the cases, the capsules did not open during the tests and were intact from a macroscopic point of view. On the other hand, when applying the coating from the 10% formulation to AAP capsules, no significant amount of drug was detected in the first two hours, while a rapid and complete release was observed in the intestinal-like fluid.

The results obtained with both types of Eudragit were surprising. While differences at pH 6.8 were expected due to the distinct features of the polymers, the outcomes at acidic pH were less predictable. In theory, both polymers should guarantee acid resistance. However, these results were always achieved using the highest polymer concentration in the presence of the lower solubility drug (AAP) or using EuS100. Interestingly, Fülöpová et al. [17] presented work on the coating of capsules using an immersion procedure. The authors evaluated EuS100-coated capsules filled with caffeine. Despite the application methods and polymer solution concentration being very similar with those of the present work, their coated capsules systematically failed the release test in the acid environment. Some results were obtained also using a formulated mixture of methacrylate-based polymers (Acryl-EZE). From the other side, the coated hypromellose capsules using a standard drum coating equipment seemed to provide different results. In fact, Fu et al. [21] reported gastro-resistance in capsules coated with different types of polymers, among which also EuSL100, although only disintegration and acid uptake tests were carried out. Apparently, in the work of Fu et al., the capsules were empty so that any influence of the excipients or APIs could not be hypothesized.

#### 2.2.2. Capsules Coated with Hypromellose Acetate Succinate Polymers

**Figure 4:**
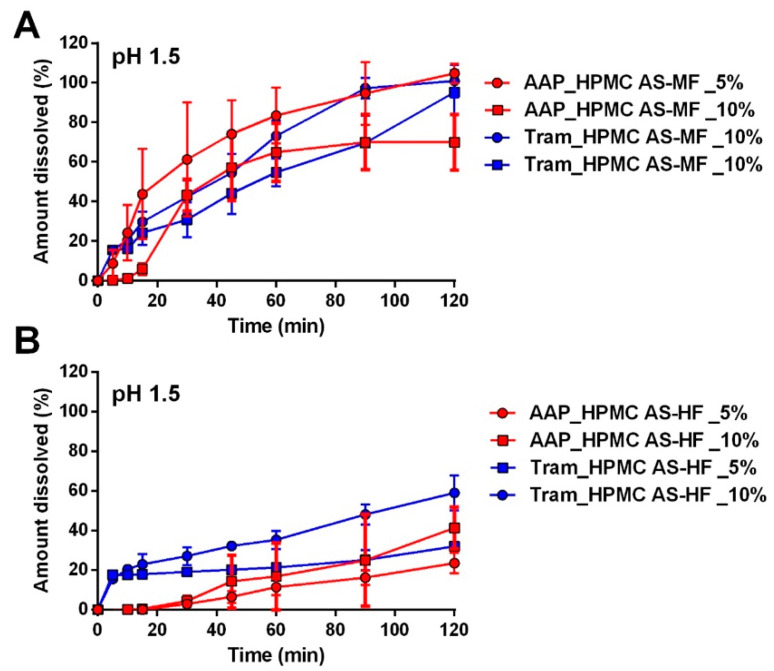
Release profiles of capsules containing AAP or Tram coated with HPMC AS-MF (**A**) and HPMC AS-HF (**B**).

The same analysis was conducted on AAP and tramadol capsules coated with the two grades of hypromellose acetate succinate, HPMC AS-MF and HPMC AS-HF (Figure 4A and B, respectively). In all cases, regardless of the drug type, polymer amount, or grade, the capsules demonstrated drug release in the acidic medium, indicating the failure of these formulations in ensuring the enteric release of the APIs. Upon comparing the dissolution results of all the coated capsules, the best performance was observed with HPMC AS-HF applied from a 10% polymer dispersion, although it did not meet the EP requirements. Once again, the role of drug solubility is evident; the amount of drug released from capsules with the same coating is consistently higher in the presence of Tram, even if only when the coating provides a certain gastro-resistance (HPMC AS-HF).

HPMC AS films are commonly used for the preparation of enteric-protected solid oral dosage forms. Fu et al. [22] reported gastro-resistance also for capsules coated with HPMC AS grades M and H, even if only through disintegration and acid uptake tests. Interestingly, HPMC AS grade H gave the same acid uptake as EuL100, while grade M reported the worst performance although also within the acceptance limits. So, the rank in term of acid resistance suggested by those authors is comparable with the results of our work, also taking into account that gastro-resistance was obtained only with EuL100 at the highest concentration and AAP as API.

HPMC AS films are commonly employed in the formulation of enteric-protected solid oral dosage forms, such as tablets or pellets. The existing literature suggests that the successful achievement of enteric formulations is dependent on the thickness of HPMC AS films [22]. To investigate and validate this hypothesis, a double coating (DC) was applied to the capsules. The DC process, involving two coating applications with a 10% polymer solution, was chosen to prevent an excessive increase in the viscosity of the polymer solution. As a result, the DC procedure led to a notable increase in the weight of the capsules, from approximately 2% to 6–8% and, consequently, an augmentation in the coating thickness. However, despite the enhancement in gastric resistance (see Figure S1), it still falls short of achieving the required values (less than 10% drug release in an acidic medium). Therefore, it is likely that an even greater quantity of polymers would need to be added, although such an approach may not be practically viable in immersion coating due to the excessively long time required for coating capsules.

**Figure 5:**
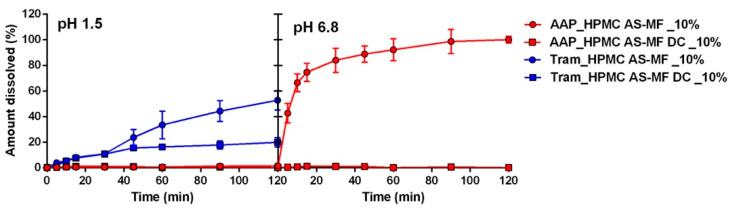
Release profiles of hypromellose capsules containing AAP or Tram coated with HPMC AS-MF single and double (DC) coating.

The inadequacy of HPMC AS in capsule coating could also be attributed to other factors, such as the insufficient adherence of the coating to the capsule surface. Typically, coatings are applied to tablets or pellets with a microscopic rough surface, facilitating the adhesion process. In contrast, capsules have a much smoother surface, potentially complicating the adhesion process. Additionally, a high interfacial tension could impede the uniform spreading of the polymer solution on the gelatin surface, resulting in uneven film formation. To validate this hypothesis, it was decided to use hypromellose capsule shells instead of gelatin. The hypromellose capsules were filled with the identical formulation as the gelatin capsules, incorporating the same APIs and excipients. Subsequently, they were coated with film solutions containing HPMC AS-MF at 10% and 10% DC. The drug dissolution profile (Figure 5) seems to confirm the hypothesis, as formulations containing AAP in hypromellose capsules do not release the API in the gastric medium when coated with a film containing 10% and 10% DC of HPMC AS-MF. However, the same phenomenon does not occur with capsules containing Tram, underscoring once again that the drug’s solubility directly influences the performance of the film coating.

#### 2.2.3. Commercial Capsules for Enteric/Delayed Release

**Figure 6:**
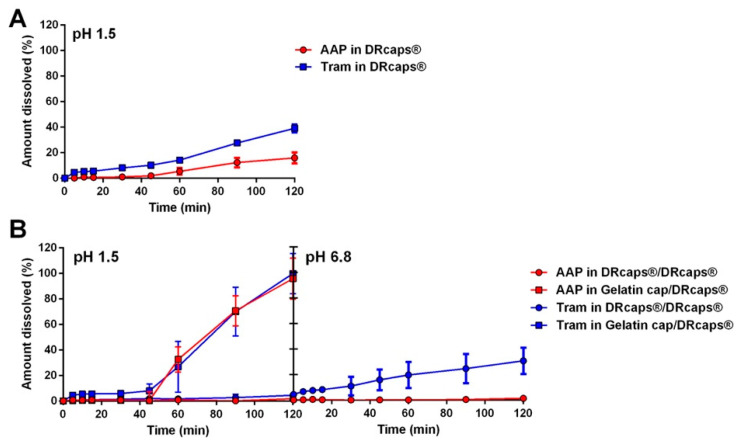
Release profiles of DRcaps^®^ capsules containing AAP or Tram (**A**) and of different combinations of DRcaps^®^ in the cap-in-cap method (**B**).

An alternative approach to preparing gastro-resistant capsules involves the use of capsule shells made from specific polymers designed to ensure release in the stomach. DRcaps^®^ are delayed release capsules composed of hypromellose and Gellan gum. The manufacturing company guarantees a release retention for about 45 min, and they are evidently not intended for the preparation of gastro-resistant capsules. Nevertheless, they are at times employed for this purpose in galenic preparations. More frequently, in the compounding of medicines within pharmacy laboratories, they are utilized to prepare gastro-resistant capsules using the cap-in-cap approach—where a filled DRcaps^®^ is placed within a larger DRcaps^®^. In this manuscript, both single and cap-in-cap DRcaps^®^ were evaluated using the same formulations employed for the coated capsules. As expected, the single DRcaps^®^ did not prevent any drug release in the gastric environment for the required time (Figure 6A). When using a highly soluble API like Tram, the DRcaps^®^ was also unable to ensure delayed release; instead, it exhibited a sustained release pattern following an almost zero-order kinetic. On the other hand, when using AAP, the release results were in line with the indications provided by the DRcaps^®^ manufacturer. The cap-in-cap approach yielded completely different results (Figure 6B). The dissolution of AAP in the acid medium and in the phosphate buffer was almost negligible, indicating that using one DRcaps^®^ inside another completely prevented AAP release for at least 4 h. Conversely, when employing a highly soluble drug like Tram, the cap-in-cap approach provided gastro-protection without preventing release in the basic environment. However, at pH 6.8, the dissolution rate of tramadol was very slow, and after 2 h, the amount of dissolved drug was below 30%. Additional attempts to achieve enteric release of the drug was carried out by placing a standard gelatin capsule inside a DRcaps^®^ (Figure 6B). Once again, no proper gastro-resistant capsules were obtained. In those cases, the dissolution kinetics resembled that of delayed release dosage forms, with a lag time of approximately 45 min, independent of the drug solubility. The latest result indicates that if the capsule shell is not in direct contact with the powder, the release rate is almost entirely controlled by the external capsule, in this case, a DRcaps^®^.

**Figure 7:**
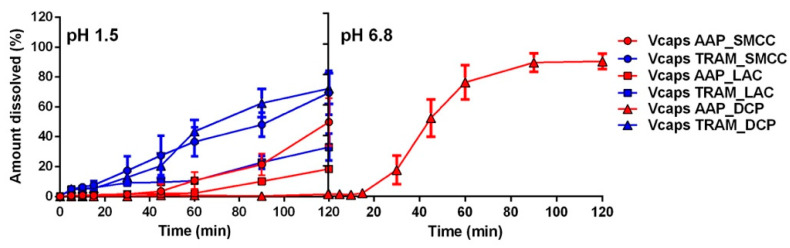
Release profiles of Vcaps capsules containing AAP or Tram as API and silicified microcrystalline cellulose (SMCC), anhydrous lactose (LAC), and anhydrous dibasic calcium phosphate (DCP) as filler.

The other type of commercial capsules evaluated were the Vcaps Enteric^®^. These capsule shells are composed of a mixture of HPMC and HPMC AS, aiming to ensure a pH-dependent release of the API due to the presence of a polymer with pH-dependent solubility (HPMC AS). The dissolution tests were initially carried out on Vcaps filled with the same formulations used for all the previous evaluations, mixtures of silicified MCC with AAP or Tram. The results were surprising (Figure 7), with the API beginning to be released in the first 45 min, in accordance with the drug solubility. However, the behavior of these capsules differed from that observed in the previous tests, as they were characterized by an unusual and unexpected event: after a short period of time in the acid medium, a gradual separation of the capsule’s cap from the body occurred, reaching the point where the two parts of the capsule shell appeared completely separated in most of the capsules tested. The failure of Vcaps with several APIs was reported by Moghrabi and Fadda [14]. The authors attributed these results to different deformation rates of the cap and body, leading to a certain opening at the junction of these two pieces. On the other hand, successful enteric release with Vcaps was reported for Octreotide acetate [23] and ketoconazole [24], although using very particular formulations such as spray-dried microparticles and amorphous solid dispersions (prepared with other enteric polymers).

From our analysis as well as from those reported in the literature, it cannot be conclusively determined whether the cap and body separation is intrinsically due to the design of Vcaps or if it is related to some defect in the batch used; however, it is likely that the presence of swelling materials inside the capsule may contribute to their opening. The formulation used for capsule filling was composed of 86.5% SMCC, an excipient consisting of microcrystalline cellulose and colloidal silicon dioxide. This material is insoluble in water, but when in contact with an aqueous phase, it absorbs water and swells. Any capsule shell cannot completely prevent the diffusion of water, and in the presence of swellable materials, an increase in internal pressure is expected. To prove this hypothesis, it has been decided to test different formulations where the SMCC was substituted with anhydrous lactose (LAC) or anhydrous dibasic calcium phosphate (DCP). LAC is a compound soluble in water but is unable to swell when in contact with it, while DCP is practically insoluble in water and does not exhibit swelling when in contact with water. The dissolution data (Figure 7) showed a slight improvement in the presence of LAC, with a lower amount of dissolved API compared to that released with the formulation containing SMCC. However, it was still possible to observe the separation of the capsule’s cap from its body. No macroscopic differences were observed in the separation process, and the lower release rate of the two drugs is very likely due to the solubility of the excipient. In fact, the presence of a highly soluble compound slows down the API dissolution process as both compete for water. In the presence of DCP, the dissolution profile exhibited a drastic improvement, although an enteric release was possible only for the less soluble drug, namely AAP (Figure 7). These results do not allow for an exact definition of the relevance of swelling; however, they certainly highlight the role of compound solubility in capsules designed for enteric release. The presence of materials capable of interacting with water represents a significant factor in the possible failure of the formulation. The pivotal influence of certain physicochemical properties of APIs and excipients was previously reported by Moghrabi and Fadda [14]. The results reported here complement their analysis, contributing to providing a much more defined picture of the real applicability of Vcaps.

Finally, we need to report that very recently Evonik industries launched a new platform of ready-to-fill capsules: EUDRACAP™ [25]. The platform is constituted by hypromellose capsules coated with Eudragit polymers, and according to the results of the present manuscript, they could represent ideal solutions for preparation of enteric-coated capsules. At the moment, there are no published independent results to support (or discourage) the use of EUDRACAP.

## 3. Materials and Methods

### 3.1. Materials

Paracetamol and tramadol HCl (both supplied by Janssen Pharma) were chosen as model drugs. Methacrylic acid–methyl methacrylate copolymers (Eudragit S100 and L100) were from Evonik Industries (Essen, Germany). Hypromellose acetate succinate (MF and HF grades) were obtained from Shin-Etsu Chemical (Tokyo, Japan). Silicified microcrystalline cellulose (Prosolv sMCC90, JRS Pharma, Rosenberg, Germany), anhydrous lactose (SuperTab 24AN, DFE Pharma, Goch, Germany) anhydrous dibasic calcium phosphate (DI-CAFOS A 60, Budenheim, Budenheim, Germany), and Triethyl Citrate (RoFarma Italia, Gaggiano, Italy) were used as received. Isopropanol (Carlo Erba, Cornaredo, Italy) and Yellow Eosin (Carlo Erba, IT) were of standard chemical grades. Gelatin capsules were purchased from ACEF (Fiorenzuola D’Arda, Italy), while HPMC cps (size 0), DRcaps^®^ (size 2 and size 0), and Vcaps^®^ Enteric capsules (size 0) were obtained from Lonza (Lonza Capsules & Health Ingredients, Basel, Switzerland).

Throughout the text, the following abbreviations are used: paracetamol (AAP), tramadol HCl (Tram), Eudragit S100 (EuS100), Eudragit L100 (EuL100), hypromellose acetate succinate MF (HPMC AS-MF), hypromellose acetate succinate HF (HPMC AS-HF), Triethyl Citrate (TEC), Prosolv sMCC90 (SMCC), SuperTab 24AN (LAC), DI-CAFOS A 60 (DCP), and isopropanol (Iso).

### 3.2. Blend Preparation and Capsule Filling

Powder blends were prepared with a composition of 13.5% active pharmaceutical ingredient (API), either AAP or TRA, and 86.5% excipients (SMCC, LAC, or DCP), using a V-shaped mixer (Laboratori Mag divisione Artha, Garbagnate Milanese, Italy) operating at 50 rpm for 5 min. After preparing the blends, capsules were filled using a semi-automatic machine (FLY 120 NEW, AD Pharm, San Vendemiano, Italy) capable of filling up to 120 capsules. Each capsule contained 0.035 g of API and 0.225 g of excipients. All prepared capsules underwent evaluation for weight uniformity, and any capsules with a variation exceeding 10% (according to EP standards) of the theoretical weight were excluded from the study. A summary of capsules content is reported in Supplementary Table S1.

### 3.3. Coating Solution Formulation

A preliminary analysis was carried out by immersing capsule shells in 50 mL mixtures composed of various iso–water ratios for 30 s to assess their resistance. The immersion duration was chosen in accordance with the recommendations of the coating equipment manufacturer. The maximum allowable water content was 8% (higher values determined capsule deformation), leading to the utilization of the iso–water ratio of 92:8 in formulating the coating solutions. The final formulations were prepared in a mixture of iso–water 92:8, employing four different polymers—EuS100, EuL100, HPMC AS-MF, and HPMC AS-HF—at concentrations of 5% and 10%. TEC was utilized as a plasticizer at a concentration of 10% with respect to the weight of the polymers. Yellow Eosin at 0.05% was added for coloration of the coating. The composition of all formulations is detailed in Table 1.

### 3.4. Film Solution Rheology

The viscosity of the coating solutions, as outlined in Table 1, was assessed (*n* = 3) using a rotational rheometer (Kinexus Lab+, Malvern, UK) equipped with a C40/4 geometry. The analysis involved incrementally increasing the shear rate from 1 to 150 s^−1^ and measuring the shear stress at 25 °C. The gathered data were graphically represented as a plot of shear rate versus stress and subsequently analyzed using a power law model:

σ = PLV γ^^PLI^ (1)

where g is the shear rate, s is the yield stress, PLV is the power law viscosity (or consistency index), and PLI is the power law index (or flow index). The power law model is able to describe the flow behavior of Newtonian, pseudoplastic, or shear-thickening samples.

### 3.5. Capsule Coating

The capsules were coated using ProCoater^®^ (Torpac, NL) equipment. This instrument employed a dip-based method for coating capsules or oblong tablets. The coating process was carried out according to the manufacturer’s instructions. In brief, the capsules were positioned in the holder and subsequently immersed in the coating solution for 30 s. Following immersion, excess liquid was removed by scrolling, and the holder with the capsules was then placed in an oven at 40 °C for 20 min. At the end of this step, only one half of each capsule was coated. The same procedure was repeated by overturning the holder to coat the other half of the capsules, ensuring complete coverage of the entire shell. This process was repeated for all the coating solutions in Table 1, resulting in the preparation of 40 coated capsules for each formulation. Images of the initial capsules as well as of capsules after the two coating steps are reported in Supplementary Figure S2.

### 3.6. Dissolution Studies

Dissolution tests was carried out using a USP dissolution apparatus type I at 50 rpm (AT7 Smart, Sotax, Nordring, Switzerland), according to the EP [26]. The dissolution procedure for the capsules involved two consecutive steps:

- The dosage forms were positioned in the basket within the vessel, where a liquid simulating stomach conditions (pH 1.5) was previously introduced and heated to 37 °C. In this medium, the capsules were expected to remain stable without releasing the API; the maximum allowable drug release was set at 10% or less [26] over a 2 h period.
- Immediately following the first step, the basket containing the capsules was transferred to a vessel filled with a liquid simulating intestinal fluids (pH 6.8) and left for an additional two hours.

Samples (1 mL) of the dissolution medium were collected using a syringe with 0.45 μm filters (Minisart syringe filter, sartorius, DE) at specific intervals during the dissolution tests:

- Gastric-like medium (minutes): 5-10-15-30-45-60-75-90-105-120;
- Intestinal-like medium (minutes): 5-10-15-30-45-60-90-120.

The drug content in these samples was determined using UV spectroscopy (UV-1800 Shimadzu, Kyoto, Japan) at wavelengths of 242.5 nm for AAP and 271 nm for Tram. Preliminarily, a calibration curve at the two wavelengths was built for both the API in the concentration range of 0.005–0.042 mg/mL (the coefficients of determination r^2^ were 0.998 and 0.999 for AAP and Tram, respectively).

Each formulation underwent assessment in triplicate at a minimum.

## 4. Conclusions

This work provides insight into the current options for personalized administration using capsules of APIs requiring enteric release. The formulation of successful enteric coating capsules using immersion methods requires a deep understanding of the properties of APIs and particularly of the enteric polymers. The success of the formulations is strongly related to the solubility of the drug, and the type and amount of polymer included are crucial elements as well. From this perspective, the use of Eudragit S100 is strongly suggested since it assures high robustness of the process, allowing the obtaining of an enteric preparation without a complex development phase.

On the other hand, the use of commercially available capsules does not appear to be an easier or feasible option. The cap-in-cap technique with DRcaps^®^ proved inadequate to guarantee an enteric release of the drugs, and the use of a single DRcaps^®^ was insufficient to prevent the release of the API in the gastric-like medium. Vcaps Enteric were able to produce enteric release dosage forms, but their performance appears strongly related to the characteristics of the active ingredient and excipients used.

## Figures and Tables

**Table 1:** Coating solution compositions.

Component	5% Polymer Formulation	10% Polymer Formulation
	Amount (%) *	Amount (%) *
Polymer **	5	10
TEC	0.5	1
Yellow Eosin	0.05	0.05
Iso–water (92:8) mixture	94.45	88.95

* Percentage respect to the total solution weight. ** EuS100, EuL100, HPMC AS-MF, and HPMC AS-HF.

## Data Availability

Data is contained within the article and Supplementary Material.

## Supplementary Materials

The following supporting information can be downloaded at: https://www.mdpi.com/article/10.3390/ph17040433/s1, Figure S1: Release profiles of capsules containing AAP or Tram double coated (DC) with HPMC AS-MF and HPMC AS-HF; Figure S2: Images of capsule before, during and after the coating process. (A) un-coated capsules; (B) Capsules after the first step of coating and drying (coating of the lower half, that is the body and part of the cap); (C) Capsules after the second step of coating and drying (coating of the higher half, that is the cap and part of the body); (D) Coated capsules removed from the holder; Table S1: Capsule content for each type of type of enteric capsule system tested.
