# Supplementary material for: Exploring Immersion Coating as a Cost-Effective Method for Small-Scale Production of Enteric-Coated Gelatin Capsules
Source: Pharmaceuticals (Basel). 2024 Mar 28;17(4):433. doi: 10.3390/ph17040433 (PMC11054423; doi:10.3390/ph17040433)
Supplement: Supplementary file 1 [file pharmaceuticals-17-00433-s001.zip › pharmaceuticals-2911879-supplementary.pdf]

Supplementary materials

of

Exploring Immersion Coating as a Cost-Effective Method for Small-Scale Production of Enteric-Coated Gelatin Capsules

Beatrice Sabbatini, Diego Romano Perinelli, Giovanni Filippo Palmieri, Marco Cespi * and Giulia Bonacucina

School of Pharmacy, University of Camerino, Camerino 62032, Italy;
beatrice.sabbatini@unicam.it (B.S.); diego.perinelli@unicam.it (D.R.P.);
gianfilippo.palmieri@unicam.it (G.F.P.); giulia.bonacucina@unicam.it (G.B.)
* Correspondence: marco.cespi@unicam.it

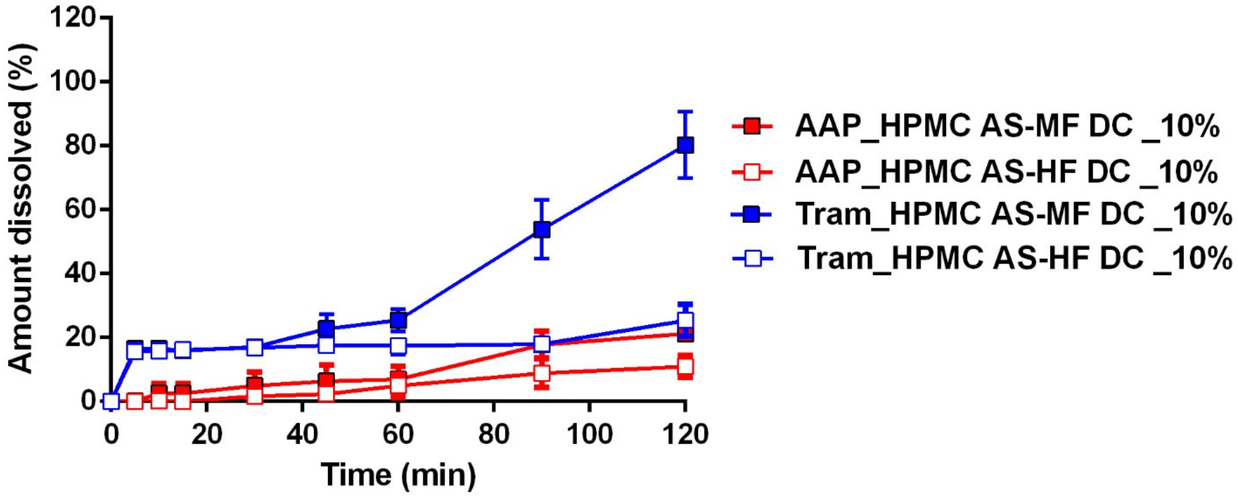

Figure S1. Release profiles of capsules containing AAP or Tram double coated (DC) with HPMC AS-MF and HPMC AS-HF.

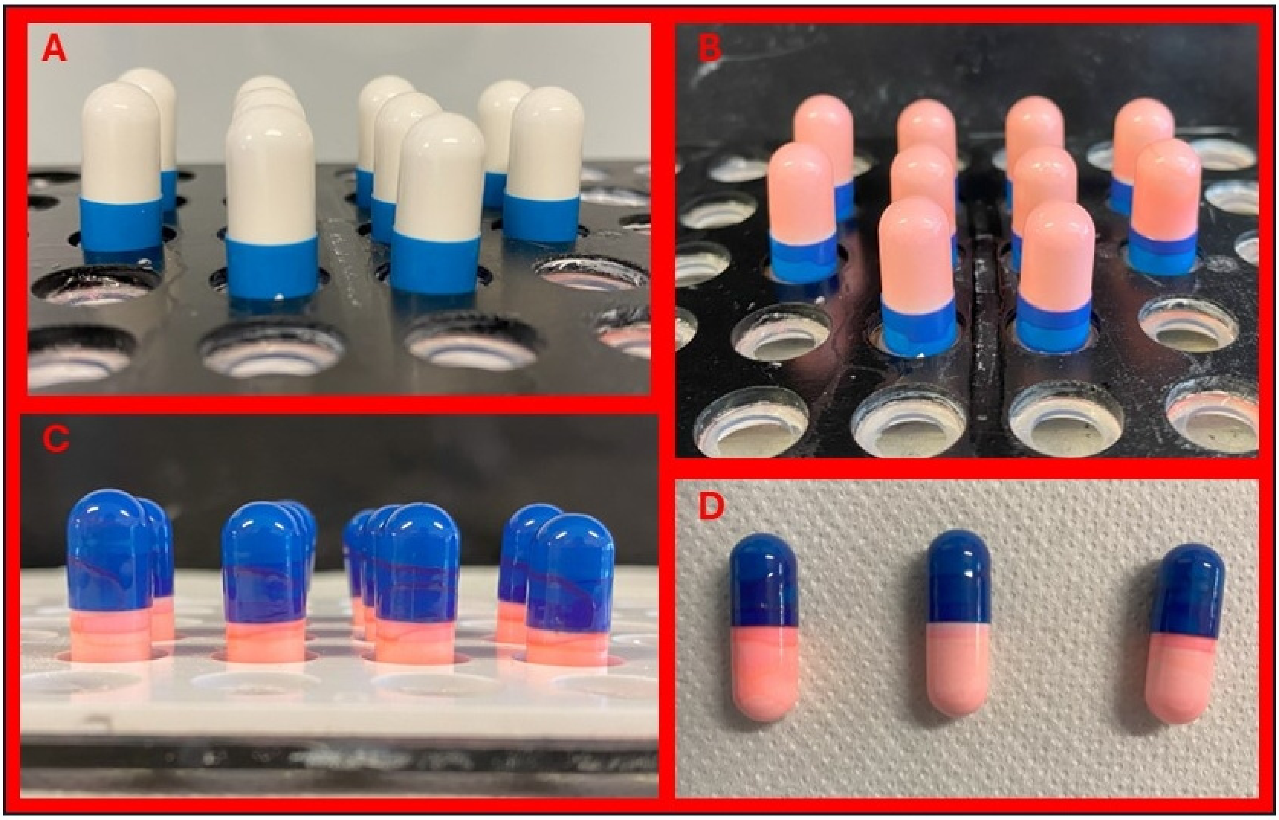

Figure S2. Images of capsule before, during and after the coating process. A) un-coated capsules; B) Capsules after the first step of coating and drying (coating of the lower half, that is the body and part of the cap); C) Capsules after the second step of coating and drying (coating of the higher half, that is the cap and part of the body); D) Coated capsules removed from the holder.

Table S1. Capsule content for each type of type of enteric capsule system tested.

**Tested capsules**	**Capsules content**	
	API (35 mg)	Excipients (225 mg)
Un-coated capsules	AAP	SMCC
Un-coated capsules	Tram	SMCC
AAP_EuS100	AAP	SMCC
Tram_EuS100	Tram	SMCC
AAP_EuL100	AAP	SMCC
Tram_EuL100	Tram	SMCC
AAP_HPMC AS-MF	AAP	SMCC
Tram_HPMC AS-MF	Tram	SMCC
AAP_HPMC AS-HF	AAP	SMCC
Tram_HPMC AS-HF	Tram	SMCC
AAP in DRcaps®	AAP	SMCC
Tram in DRcaps®	Tram	SMCC
AAP in DRcaps®/ DRcaps®	AAP	SMCC
Tram in DRcaps®/ DRcaps®	Tram	SMCC
AAP in gelatin caps/ DRcaps®	AAP	SMCC
Tram in gelatin caps/ DRcaps®	Tram	SMCC
Vcaps AAP_Lac	AAP	LAC
Vcaps Tram_Lac	Tram	LAC
Vcaps AAP_SMCC	AAP	SMCC
Vcaps Tram_SMCC	Tram	SMCC
Vcaps AAP_DCP	AAP	DCP
Vcaps Tram_DCP	Tram	DCP
